# Cosegregation of asymmetric features during cell division

**DOI:** 10.1098/rsob.210116

**Published:** 2021-08-04

**Authors:** Silje Anda, Erik Boye, Kay Oliver Schink, Beata Grallert

**Affiliations:** ^1^ Department of Radiation Biology, Oslo University Hospital, Oslo, Norway; ^2^ Department of Molecular Cell Biology, Institute for Cancer Research, Oslo University Hospital, Oslo, Norway; ^3^ Department of Biosciences, University of Oslo, Oslo, Norway

**Keywords:** cosegregation, asymmetric segregation, spindle pole body, DNA segregation

## Abstract

Cellular asymmetry plays a major role in the ageing and evolution of multicellular organisms. However, it remains unknown how the cell distinguishes ‘old’ from ‘new’ and whether asymmetry is an attribute of highly specialized cells or a feature inherent in all cells. Here, we investigate the segregation of three asymmetric features: old and new DNA, the spindle pole body (SPB, the centrosome analogue) and the old and new cell ends, using a simple unicellular eukaryote, *Schizosaccharomyces pombe*. To our knowledge, this is the first study exploring three asymmetric features in the same cells. We show that of the three chromosomes of *S. pombe*, chromosome I containing the new parental strand, preferentially segregated to the cells inheriting the old cell end. Furthermore, the new SPB also preferentially segregated to the cells inheriting the old end. Our results suggest that the ability to distinguish ‘old’ from ‘new’ and to segregate DNA asymmetrically are inherent features even in simple unicellular eukaryotes.

## Graphical abstract

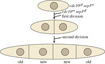

## 1. Introduction

Asymmetry plays a key role in both uni- and multicellular organisms, establishing a wide range of features from uneven distribution of ageing factors between mother and daughter cells in *Saccharomyces cerevisiae* to the establishment of separate cell lineages during development in multicellular organisms [1]. Two not mutually exclusive hypotheses have been proposed to explain the phenomenon of asymmetric cell division: one suggests that asymmetry is imposed by the cells' exposure to the extracellular environment; the other postulates that the information for cellular asymmetry is found internally in the cell. Intriguingly, multicellularity has arisen several times in the course of evolution [2], and asymmetric cell divisions are prerequisite for most forms of multicellularity (when it involves specialization). This favours the hypothesis that ancient organisms all share a molecular machinery that inherently provides a basis for asymmetric cell division [3].

Asymmetric segregation of a number of subcellular constituents has been reported, including centrosomes, proteins, organelles and nucleic acids [4–6]. Specifically, asymmetric DNA segregation has been described in several organisms. For example, sister chromatid segregation is not random in *Escherichia coli* [7], a subpopulation of mouse adult skeletal muscle cells segregates DNA strands asymmetrically [8] and X and Y chromosomes are non-randomly segregated in *Drosophila* [2]. The question of asymmetric DNA segregation came into the limelight by the ‘immortal strand hypothesis’, which postulates that asymmetric DNA segregation is a mechanism for stem cells to protect their genome from accumulating mutations occurring during replication [9]. Even though a number of studies support asymmetric DNA segregation, just as many reports argue against it (e.g. [10–12]). A demonstration of asymmetric sister chromatid segregation is technically challenging and even separate studies on the same type of tissues have provided opposite results [13–17]. This has led to a long-standing debate about both the occurrence and mechanisms behind asymmetric DNA segregation [18,19].

Asymmetric DNA segregation requires that the cells are able to keep track of old and new DNA strands through cell divisions. In order to address the question whether asymmetric DNA segregation is an ancient feature, we employed an approach that allowed us to reliably follow DNA segregation through two generations in a simple and not very specialized model organism, fission yeast.

Fission yeast is a unicellular eukaryote that displays several asymmetric traits. First, an obvious feature that could generate asymmetric markers within a fission yeast cell is the fact that one end is produced by the last cytokinesis, whereas the other persists from the preceding cell cycle. Consistently, the growth of the cells is clearly asymmetric, with one of the ends, the old end, starting to grow after mitosis and the other end, the new end, starting to grow at about the time of completion of S phase [20]. Cell growth is determined by polarity markers at the ends of the cells as well as the microtubule cytoskeleton [21].

Another asymmetric feature in fission yeast cells is the centrosome equivalent, the spindle pole body (SPB). The SPB is a microtubule-organizing centre which is duplicated at the G1/S transition and contains asymmetric components. The SPB is the centre of a conserved signalling pathway called the septum initiation network (SIN), which is involved in the regulation of cytokinesis [22–24]. Activation of the SIN always occurs on the new SPB [25–27], but the significance of this asymmetry for any differences between the daughter cells or the asymmetric growth pattern is not clear.

One of the best known asymmetric features in fission yeast is the mating type of the cells, which was shown to be regulated by epigenetic differences between sister chromatids [28,29]. Whether or how these are related to other asymmetric features in the cells is not known. Notably, this mechanism of mating-type switching provided the basis for one of the models for non-random DNA segregation: the strand-specific imprinting and selective chromatid segregation model (SSIS), which suggests that epigenetic differences between sister chromatids may drive selective sister chromatid segregation [30].

We have designed an experimental system to study three asymmetric features in fission yeast. Using halogenated thymidine analogues we employed a double-labelling technique that allowed us to specifically label the DNA made in each S phase, making it possible to distinguish old from new DNA strands. In addition, we followed the segregation of an SPB component that allowed us to identify old and new SPBs, and we also observed the asymmetric growth pattern in the same cells. We observe cosegregation of the new SPB with the old cell ends and a non-random segregation of old and new chromosome I. These results suggest that the potential for asymmetric DNA segregation and distinguishing old from new are ancient features present even in a simple unicellular organism.

## 2. Results

### 2.1. Tracking progeny for two generations

**Figure 1.**
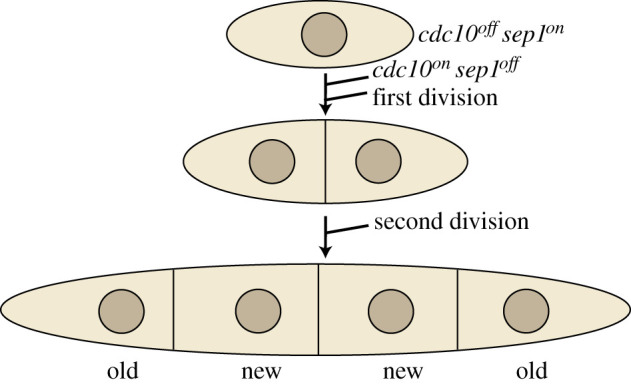
Strategy to identify granddaughter cells deriving from a single G1-arrested grandmother cell. See manuscript text for explanation.

In order to observe the segregation pattern of the DNA as well as the segregation pattern of the SPBs it is essential to be able to identify the daughter cells for at least two cell cycles. Fission yeast daughter cells are morphologically very similar, thus the need for a reliable system in which to follow cell segregation is essential. We exploited a *sep1* mutant that is defective in cytokinesis and forms mycelia with the daughter cells staying unseparated [31]. After two generations four-celled mycelia are formed, in which the asymmetric growth pattern can be observed [31]. The outermost cells in the four-cell mycelia inherit the oldest cell ends and will be referred to as ‘old’ cells, while the inner two cells inherited the new ends and will be referred to as ‘new’ cells (figure 1). After the four-celled stage, the mycelia break up as a consequence of the asymmetric growth pattern [31]. Therefore, a *sep1* culture consists of a mixture of single cells, doublets and mycelia of three or four cells. To start our experiments with a homogeneous population of cells we generated a conditional *sep1* mutant by fusing the *sep1* gene to the hormone-binding domain of the oestrogen receptor [32], enabling us to regulate the activity of the Sep1 protein by growing the cells in the presence (Sep1 on) or absence (Sep1 off) of oestradiol. To be able to synchronize the cells in G1 phase we introduced a temperature-sensitive *cdc10* mutation. Using this strain the fate of the chromosomes and the SPBs can be followed in single cells released from G1 and proceeding to generate mycelia with four granddaughter cells (figure 1).

### 2.2. Following DNA segregation over two generations

To be able to follow the segregation of chromatids with new or old parental DNA strands, we exploited the halogenated thymidine analogues IdU and CldU or EdU. *Schizosaccharomyces pombe* cells do not take up and incorporate exogenous thymidine analogues naturally; therefore, the human equilibrative nucleoside transporter gene (hENT1) and the herpes simplex virus thymidine kinase gene (hsv-tk) were introduced [33].

**Figure 2.**
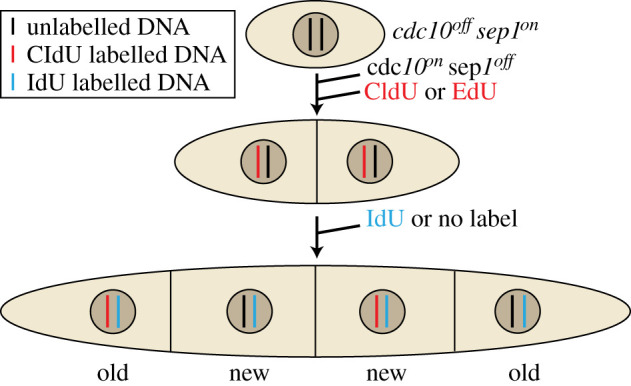
Experimental outline for following DNA segregation for two generations. See manuscript text for explanation.

The cells were first arrested in G1 phase by growth under restrictive conditions for the temperature-sensitive *cdc10* mutation (figure 2). At the same time, Sep1 was kept active by the presence of oestradiol (Sep1 on). Sep1 was then inactivated by washing out the oestradiol and the cells were shifted to the permissive temperature for *cdc10*, releasing them into S phase. To specifically label the DNA strands synthesized in the first generation, CldU was added during the G1 arrest and kept in the medium until the first S phase was completed. The cells were then washed with medium to remove CldU. Before the following S phase, IdU was added to label the DNA strand replicated in the second generation. After the second S phase was completed, IdU was removed from the medium. The timing was determined in preliminary experiments using DNA staining in flow cytometry. When septa were formed, the cells were fixed and processed.

In the second generation, each chromosome will contain one strand newly synthesized in the second S phase and one strand that was either an original template (old parental DNA strand; black in figure 2) or a newly synthesized template (new parental DNA strand; red in figure 2). Thus, the segregation pattern of the DNA synthesized in the first S phase can be observed in the second generation. Random segregation would lead to even distribution of the first label between the daughter cells, whereas non-random segregation would lead to uneven distribution. The chromosome that contains the newly synthesized parental strand from the first S phase will be referred to as new chromosome throughout this paper.

To measure the segregation pattern of the labelled DNA, we acquired fluorescence-microscopic z-stacks of entire cells to ensure that all the fluorescence within one nucleus was captured. We then measured the integrated intensity of the labelled DNA using a sum-projected image of the z-stacks.

**Figure 3.**
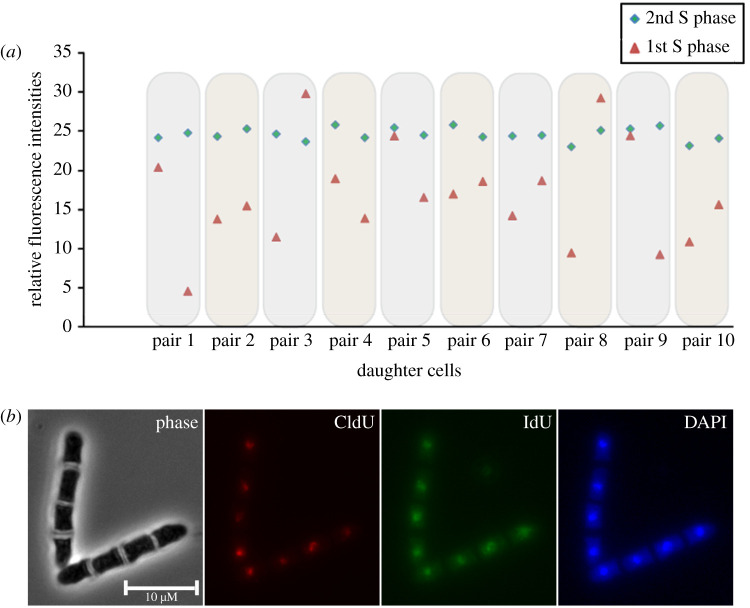
Granddaughter cells inherit a mixture of old and new DNA. (*a*) Quantification of CldU (1st S phase) and IdU (2nd S phase) incorporation in 10 cell pairs. (*b*) An example of four-celled mycelium after CldU and IdU labelling. Note the uneven distribution of CldU and even distribution of IdU. Cells were grown and labelled as outlined in figure 2.

Predictably, the distribution of the first label (CldU) was uneven between the four daughter cells, whereas the distribution of the second label (IdU) was even (figure 3). The even distribution of the second label serves as a control and it demonstrates that the incorporation of the analogue is proportional to the amount of newly synthesized DNA. Assuming that the label is distributed uniformly along each of the chromosomes, we can determine the DNA segregation pattern based on the fluorescence intensities of the analogue-labelled DNA. Thus, the uneven distribution of the first label allows us to conclude that the daughter cells inherited unequal amounts of old and new DNA.

A caveat in any experiment using thymidine analogues is their toxicity and mutagenicity [34,35]. Such analogues have been shown to induce checkpoint responses and arrest the cell cycle as well as to increase sister chromatid exchange (SCE) at high concentrations [36–40]. In order to minimize the effect the analogue had on the cells, we opted to only label the first S phase in subsequent experiments and selected EdU, which can be used at lower concentrations than halogenated analogues and disturbs cell-cycle progression to a lesser extent [35].

### 2.3. Biased chromosome segregation

**Figure 4.**
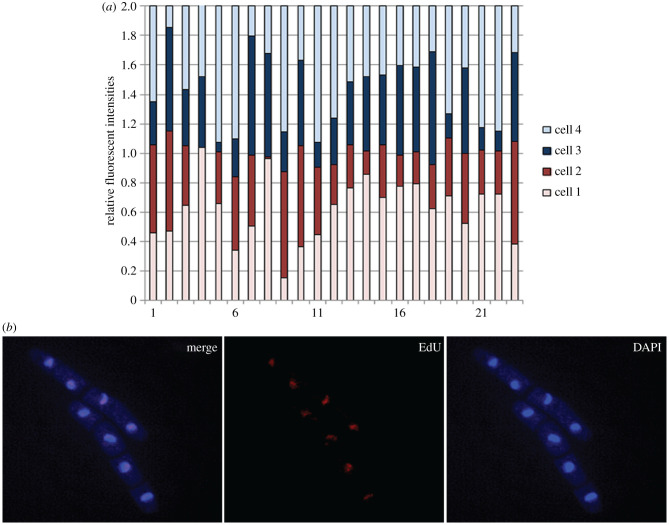
EdU labelling in the first S phase. (*a*) Relative fluorescence intensities of the EdU-labelled DNA in each of the four nuclei in four-celled mycelia. Each stacked bar represents four granddaughter cells, with the fluorescence intensities for each nucleus shown as different colours. (*b*) Microscopy images of EdU-labelled cells. Cells were grown and labelled as outlined in figure 2 and fixed and processed as described in Material and methods.

We observed that most, but not all, nuclei contained some EdU-labelled DNA, and the intensity of the label varied from nucleus to nucleus (figure 4, electronic supplementary material, table S1). The cell pairs within the four-celled mycelia had similar amounts of label (cells 1 + 2 together had similar amount of label as cells 3 + 4) (figure 4), as expected when a labelled set of chromosomes is distributed between the two daughter cells. This finding further supports the concept that the intensities can be used to follow the segregation of newly synthesized chromatids and that the quantifications are correct and warranted.

**Figure 5.**
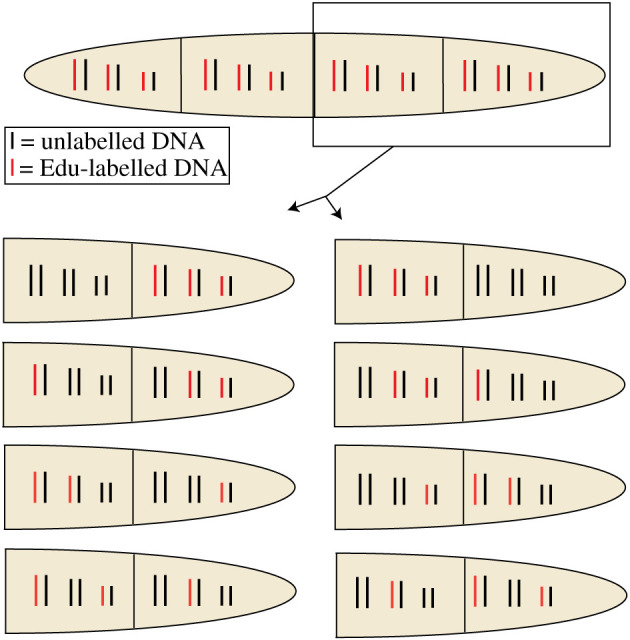
Possible segregation patterns. Possible outcomes of the segregation of the EdU-labelled DNA strands (red) synthesized during the first S phase between daughter cells. See manuscript text for details.

Since most cells inherited some labelled DNA, we conclude that there is no strict segregation of all old parental DNA and all new parental DNA to one daughter cell. However, these data do not exclude that a particular chromosome follows a non-random segregation pattern. Fission yeast has three chromosomes and the intensity of the label in each cell depends on which particular labelled chromatid(s) the cell inherited. The distribution of sister chromatids with old and new parental DNA strands between granddaughter cells can have eight different outcomes (figure 5). If sister chromatid segregation is random, all of the combinations depicted in figure 5 should occur at equal frequencies. Alternatively, some combinations may occur more frequently than others, suggesting a biased segregation. The three chromosomes of *S. pombe* have rather different sizes; chromosome I is 5.7 Mb, chromosome II is 4.6 Mb and chromosome III is 3.5 Mb [41]. The distribution of different combinations of the labelled chromosomes will result in different fluorescence intensities and can, therefore, be distinguished. Table 1 shows the fluorescence intensities that can be predicted when different combinations of the labelled chromatids segregate to a particular cell.

**Table 1.** Expected fluorescent intensities in cell 1 and 2, depending on the distribution and size of the three chromosomes.

new chromatid in cell 1	expected fluorescent intensity in cell 1	expected fluorescent intensity in cell 2	expected range of fluorescent intensity in cell 1
Chr I	0.413	0.587	0.373–0.453
Chr II	0.333	0.667	0.294–0.373
Chr III	0.254	0.746	0.214–0.293
Chr I + II	0.746	0.254	0.707–0.786
Chr I + III	0.667	0.333	0.627–0.706
Chr II + III	0.587	0.413	0.547–0.627
Chr I + II + III	1	0	0.850–1
none	0	1	0–0.08

Table 2 shows the observed distribution of the labelled chromosomes between the ‘old’ and ‘new’ cells based on the intensity measurements (electronic supplementary material, table S1). We employed strict margins (theoretical value ±0.04) (table 1) when determining which chromosomes each cell inherited, and cells displaying fluorescence intensities outside these ranges were disregarded. Ranges were determined to ensure that there are no overlaps and that there are not too many outliers. Interestingly, the observed distribution was significantly different from a theoretical random distribution (table 2). On closer inspection of the distribution of each chromosome, we found that chromosome I displays a non-random distribution between the two daughter cells and the new chromosome I preferentially, in 64% of the cell pairs, segregates to the daughter cell with the old end (table 3). By contrast, no preferential segregation of chromosomes II and III could be detected (table 3). Consistently, a previous study examining the distribution of chromosome II also concluded that it segregates randomly between the two daughter cells [42]. Similar conclusions were drawn when stricter ranges were used, such as theoretical value ±0.025 (data not shown).

**Table 2.** Distribution of chromosomes containing the new parental strand in old and new cells. *n* = 117.

new chromosome	in old cell (number of cells)	in new cell (number of cells)	*p*-value
Chr I	23	12	0.017
Chr II	12	18	
Chr III	12	24	
I + II	24	12	
I + III	18	12	
II + III	12	23	
I + II + III	10	6	
none	6	10

**Table 3.** Segregation of chromosomes between the ‘old’ and ‘new’ cells. *n* = 117.

new chromatid of	‘old’ cells (number of cells)	‘new’ cell (number of cells)	‘old’ cells (% distribution)	‘new’ cell (% distribution)	*p*-value
Chr I	75	42	64	36	0.002
Chr II	58	59	50	50	0.926
Chr III	52	65	44	56	0.229

We have also addressed whether the segregation of one chromosome correlates with that of another. While there is no strong preference for any one pair, cosegregation of all three chromosomes occurs less frequently than expected in case of random segregation (table 4).

**Table 4.** Cosegregation of chromosomes. *n* = 117.

	number of cells	%	*p*-value
Chr I and II	52	44	0.229
Chr I and III	47	40	0.033
Chr II and III	50	43	0.116
all three	16	14	0.005

### 2.4. Sister chromatid exchange as a possible problem when assessing asymmetric DNA segregation

**Figure 6.**
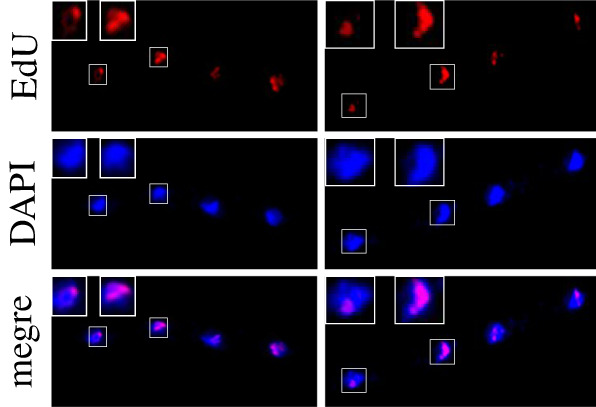
Super-resolution images of cells arrested in mitosis. Cells were synchronized and labelled as described in figure 2, then arrested in the next mitosis with carbendazim. Distribution of EdU-labelled DNA (red) was investigated by super-resolution microscopy. DNA was stained with DAPI (blue).

One concern when analysing our data is the effect SCE may have on the fluorescence intensity analyses. Extensive and numerous SCE events would lead to higher or lower fluorescence intensities than one would expect for a given chromosome, which would either shift the values outside the ranges we used or, in the worst-case scenario, shift the values to the next range and lead to false conclusions. This possibility was of particular concern upon the finding that all three chromosomes cosegregate less frequently than expected, since this is the very category that would be underrepresented due to extensive SCE. To estimate the frequency of SCE events in the course of two generations we repeated the experiment described above but arrested the cells in the third mitosis after release from the *cdc10* block by using carbendazim (CBZ), a microtubule depolymerizing drug that arrests the cells in mitosis. We visualized the EdU-labelled DNA within condensed nuclei and compared it to the DAPI-stained DNA. If SCE is extensive, we would expect that EdU-labelled regions are distributed throughout the nuclei and that the EdU-signal largely overlaps with the DAPI signal. However, if SCE events are not frequent, the EdU-labelled DNA would give strong signals confined to small localized areas within the nucleus. While this approach does not allow an accurate quantification of the amount of DNA transferred between the DNA strands, it would allow us to detect numerous and extensive SCE. We observed that the EdU-labelled DNA was confined to a few localized regions within the DAPI-stained region (figure 6). These observations suggest that the frequency of SCE events within two generations is low and thus low amounts of DNA have been exchanged.

### 2.5. Asymmetric segregation of the SPB

**Figure 7.**
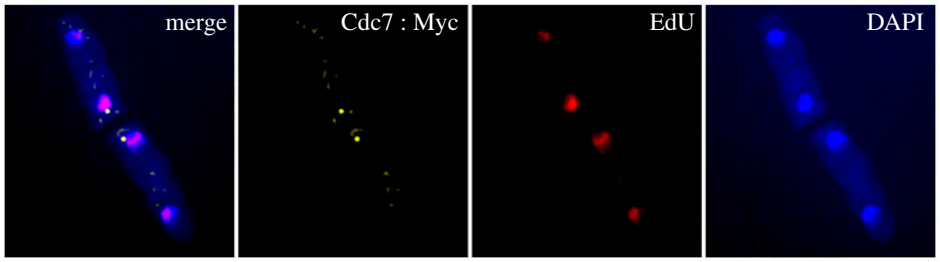
Microscopy images of cells carrying Cdc7 : myc with EdU-labelled DNA. Cells were grown in YE containing 1μM oestradiol and synchronized by lactose gradient centrifugation. Two hours after synchronization 10 µM EdU was added and kept in the medium for 1 h. The EdU and oestradiol was removed by washing three times with equal volumes of YE and the cells were incubated for 6–7 h until four-celled mycelia were formed.

During each cell cycle, the SPB duplicates and the new and old SPBs can be distinguished by their different protein compositions. One of the proteins that is differentially recruited to the old and new SPBs is Cdc7, which initially binds both SPBs in metaphase, then it leaves the old SPB and stays bound only to the new SPB until late anaphase [16]. We set out to determine whether the age of the SPB correlates with either asymmetric DNA segregation or the ‘old’ and ‘new’ cells. We used a strain carrying a Myc-tagged Cdc7. However, at 36°C many of the cells carrying this tag did not separate after cytokinesis, making it difficult to synchronize the cells with a *cdc10* block and release. Therefore, cells carrying a Myc-tagged version of Cdc7 were synchronized by lactose gradient centrifugation, the DNA was labelled as described in the previous chapters and the segregation patterns of both Cdc7 and the DNA to the ‘old’ and ‘new’ cells were determined (figure 7, electronic supplementary material, table S2).

Interestingly, the segregation of the Cdc7-bound new SPB was clearly not random and appeared in the ‘old’ cell in approximately 65% of the cells (*p* = 0.0021) (table 5). We conclude that the new SPB preferentially, but not exclusively, segregates to the ‘old’ cell and that the old SPB preferentially goes to the ‘new’ cell.

**Table 5.** The distribution of Cdc7 between the ‘old’ and ‘new’ cells. *n* = 104.

number of cells		% distribution		*p*-value
Cdc7 (new SPB) in ‘old’ cell	Cdc7 (new SPB) in ‘new’ cell	Cdc7 (new SPB) in ‘old’ cell	Cdc7 (new SPB) in ‘new’ cell	
68	36	65.4	34.6	0.0021

Having seen asymmetric segregation of both the SPB and chromosome I, an obvious question is whether these two asymmetric traits cosegregate. As shown in electronic supplementary material, table S3, the two traits segregated independently. It should be noted, however, that in the *cdc7-myc* background the biased segregation of chromosome I was not observed (electronic supplementary material, table S4). By contrast, a strain carrying Cdc7 without the tag but synchronized by lactose gradient centrifugation did display the biased segregation (electronic supplementary material, tables S5 and S6) similar to that observed in the cells synchronized by *cdc10* block and release (table 3). Together with the observation that the *cdc7-myc* strain has a slight temperature-sensitive cytokinesis defect, these findings suggest that the tag on Cdc7 confers a hypomorphic phenotype, which is responsible for the loss of the biased segregation of chromosome I.

### 2.6. Chromosome segregation is random after heat stress

In response to cellular stress, the damage to proteins and organelles might be repaired, or asymmetrically segregated to one of the daughter cells. Fission yeast cells were reported to not age under normal conditions, but they asymmetrically segregate protein aggregates, that is to say age, after stress [43]. By contrast, mild stress, such as calorie restriction or heat stress, help prevent ageing [44], probably by promoting a more equal segregation of ageing factors [45]. To address whether the biased segregation of chromosome I is affected by stress we repeated the experiment described in figure 2, but exposed the cells to a 30 min heat stress at 40°C before releasing the cells into the cell cycle. Surprisingly, the biased segregation of chromosome I was no longer observed in the cells exposed to heat stress (table 6; electronic supplementary material, table S7), nor was the cosegregation of all three chromosomes less frequent than expected (table 7).

**Table 6.** Segregation of chromosomes between the ‘old’ and ‘new’ cells after heat stress. *n* = 124.

new chromatid of	‘old’ cells (number of cells)	‘new’ cell (number of cells)	‘old’ cells (% distribution)	‘new’ cell (% distribution)	*p*-value
Chr I	55	69	48	52	0.2087
Chr II	63	61	49	51	0.8575
Chr III	51	73	41	59	0.0725

**Table 7.** Cosegregation of chromosomes after heat stress. *n* = 124.

	number of cells	%	*p*-value
Chr I and II	52	42	0.072
Chr I and III	68	55	0.281
Chr II and III	66	53	0.472
all three	31	25	1

## 3. Discussion

We have studied three asymmetric features over two generations in fission yeast cells; the segregation of DNA strands, of the SPB and old and new cell ends. We have shown that chromosome I, but not chromosomes II and III, display an asymmetric pattern in that the new chromatid preferentially segregates to the ‘old’ cells. In addition, the SPB also segregates asymmetrically between the two daughter cells, with the new SPB segregating to the ‘old’ cell with higher probability.

These observations suggest that *S. pombe* cells have the ability to distinguish between new and old DNA, suggesting that the potential for asymmetric DNA segregation is an inherent feature even in simple, unicellular eukaryotes. Due to the semiconservative nature of DNA replication, the most obvious asymmetric feature in the cell is the DNA, which carries all the genetic information and directs most events in the cell. Since sister chromatids are inherently non-equivalent both when considering DNA-strand sequences and replication history, it is tempting to speculate that the source of asymmetry might stem from the semiconservative nature of DNA replication or some mechanism closely associated with DNA replication.

Our approach makes it possible to study the segregation pattern at a single-chromosome level over only two generations in granddaughter cells and avoids several pitfalls that have been problematic when studying this phenomenon. Several studies have employed the label retention method in which cells incorporate a thymidine analogue over a prolonged period of time and after several generations, the number of label-retaining cells is scored. These studies are hampered by the severe effects that BrdU or similar thymidine analogues exert on the cell cycle [46,47] After several generations, the presence of BrdU or other analogues will impact the survival, cell-cycle progression and the growth rate of the cells [35,47]. In addition, the frequency of SCE increases with increasing concentrations of BrdU [34], skewing the results towards equal distribution between daughter cells. Another potential pitfall is that the observed label-retaining cells may result from cellular quiescence. Similarly, if the original template is not labelled because non-cycling cells are present, the results could be misinterpreted. More recent methods involve extensive BrdU-labelling of the new strand, which is then degraded and the remaining, old DNA is analysed either by sequencing (Strand-Seq) or by FISH (CO-FISH) [8,48]. Strand-Seq analyses DNA after cell lysis and DNA extraction, making it difficult to analyse cosegregation with other features. CO-FISH analyses single cells and after only two generations, but can only investigate limited regions of a chromosome at a time. Our approach involves three important advantages as compared to previously used approaches. First, it allows us to track single sister chromatids over as few as two generations. Second, we analyse single cells as opposed to a population of cells and can thus observe other asymmetric features in the same cells. Third, since thymidine analogues may affect the frequency of SCE, we minimized the amount of analogues that the cells were exposed to. We have titrated the analogues to the lowest detectable concentration [35], limited the time of exposure to S phase, and used only EdU (as opposed to double-labelling with two different analogues).

In spite of these measures, we were concerned about the possible effect of SCE on the fluorescence intensities. Extensive SCE would shift the intensities towards the average, 0.5/cell, leading to an underestimation of the real extent of asymmetry. On the other hand, isolated and not extensive SCE events might skew the observed intensities towards either higher or lower values. The following observations argue against SCE being responsible for the observed asymmetric DNA segregation. First, using high-resolution imaging we observed few and well-defined areas of EdU-labelled DNA within the DAPI-stained nuclei in CBZ-arrested cells, arguing against a high frequency of SCE, which would result in the condensed nuclei being littered with patches of EdU-stained DNA. Second, heat shock abolished both the asymmetric segregation of chromosome I and the low number of cells where all three labelled chromosomes cosegregate. In the light of this finding it is less likely that the apparent asymmetric segregation is an artefact of SCE, unless we also invoke a mechanism for SCE that is highly heat-sensitive.

Previous studies investigating asymmetric DNA segregation have focused on the ‘old’ DNA strand being selectively segregated into a particular cell type (for example mother versus daughter cell or stem cell versus differentiated cell). However, the potential for biased DNA segregation may not be associated with a particular cell type. Our approach allows revealing unequal segregation of the new chromosomes between daughters of the same cell type. No general rule could be observed for cosegregation of all the old and new DNA and the ‘age’ of the cell (based on the cell ends). However, there are several features beside the mother–daughter identity that may be connected to chromosome segregation and the individual chromosomes may be differentially segregated to mother and daughter cells. An example of differential DNA segregation is demonstrated in *Drosophila*, where the autosomes segregate randomly, whereas the sex chromosomes segregate non-randomly [2]. Similarly, in fission yeast, we observe that while chromosomes II and III segregate randomly, the new chromosome I preferentially segregates to the ‘old’ cell.

An obvious question is whether specific asymmetric traits of the cell are connected to one or more of the chromosomes or if the asymmetrically segregating chromosomes carry different epigenetic markers which direct a certain segregation pattern [49]. A well-studied example of an epigenetically determined asymmetric trait is the mating-type switching in *S. pombe*, where one in four granddaughters switches mating type [28,50]. This process is regulated by an epigenetic imprint generated during lagging-strand synthesis [51]. This developmental asymmetry is restricted to one locus and such an asymmetry is not likely to dictate asymmetric segregation of the chromosome. If asymmetric segregation of a chromosome was dictated by an epigenetic change, it would have to occur at the centromere region. Combined with epigenetic differences at several loci, this could allow the preservation of epigenetic differences within a defined cellular pedigree.

The centrosomes duplicate once per cell cycle and the daughters inherit one centrosome each. In *Drosophila melanogaster* the male germline cells retain the mother centrosome [52], and in *S. cerevisiae* the old SPB is always inherited by the bud [53]. Similarly, we found asymmetric SPB segregation in *S. pombe* in that the old SPB segregates to the ‘new’ cell in 65% of the cases. Interestingly, in a previous study segregation of the SPB appeared random [54]. The different conclusions might be explained by different tags being used in the two papers (Cdc7 : GFP versus Cdc7 : Myc) as well as different genetic backgrounds of the parent strains.

Full maturation of the SPB in fission yeast takes two generations, so that a grandmother, mother and granddaughter SPB can be discerned [25], which is reminiscent of DNA replication where also a grandmother-strand, mother-strands and daughter-strands can be distinguished. Furthermore, DNA replication and centrosome duplication are coregulated to ensure that they are initiated at the same time [55]. However, we see no correlation between the age of the SPB and the biased DNA segregation, indicating that these two asymmetric features do not cosegregate. It is important to note that in the strain carrying the tagged Cdc7 the biased segregation of chromosome I was not observed (electronic supplementary material, table S4). This might be due to a slight loss-of-function effect of the tag since we also observed that at 36°C the cells carrying the tag had a mild cytokinesis defect, suggesting that the tag renders Cdc7 temperature sensitive (data not shown). Even though we chose to synchronize these cells by size-selection rather than using a temperature-sensitive mutant to avoid a temperature shift, the tag might have conferred a hypomorphic phenotype even at low temperature. On the other hand, the fact that tagging an SPB component can affect asymmetric DNA segregation hints at a mechanism to ensure asymmetry involving the SPB.

We have provided data suggesting that the unicellular fission yeast cells can distinguish between old and new DNA and that this results in a biased distribution of chromosome I between daughter cells. Furthermore, the SPB also follows an asymmetric segregation pattern between the daughter cells. Thus, being able to distinguish ‘old’ and ‘new’ is an ancient feature in evolutionary terms.

### 3.1. Opening up

The finding that a unicellular organism has the ability to distinguish old and new DNA suggests that it is based on some inherent feature rather than a dedicated mechanism that evolved only in higher organisms. The immortal strand hypothesis postulated that asymmetric DNA segregation could serve as means to protect the template strand from replication errors. However, this requires suppression of recombination and SSC events, which would make the cells vulnerable to DNA damage. We reason that in simple organisms efficient DNA repair mechanisms provide a stronger selective advantage than maintaining selective DNA segregation over several generations.

We propose that the mechanism of asymmetric DNA segregation involves the asymmetric attachment of the sister chromatids to the mitotic spindle orchestrated by the SPB and that the interaction between the SPB and the cell cortex drives the segregation of the new chromosome I to the ‘old’ cell. In fission yeast, the new SPB is always associated with the active SIN [25]. In budding yeast, the old SPB always segregates into the bud and the new SPB remains in the mother cell [53,56], which was attributed to different interactions between the SPB and the cell cortex in the bud and the mother cell [53]. The old chromatid I in fission yeast might attract kinetochore proteins that preferentially associate with microtubules from the old SPB, simply because it might be better at nucleating microtubules. Indeed, in budding yeast asymmetric segregation of kinetochore proteins has been described, even though this was limited to the first post-meiotic lineage [57]. Differential recruitment of kinetochore proteins might be due to the semiconservative nature of DNA replication and/or epigenetic differences at the centromere. Asymmetric association of kinetochores with microtubules emanating from the old SPB would leave the new chromatid I to be captured by microtubules emanating from the new SPB. The SIN is always activated on the new SPB [26], which might direct its segregation towards the old cell end, accounting for the biased segregation of chromatid I. It should be noted that chromosome I has the smallest centromere and as such it would be most sensitive to any bias in the asymmetric loading of kinetochore proteins and/or association with spindle microtubules. In the cells exposed to heat stress chromosome, I no longer segregated in an asymmetric manner. Following the above line of reasoning, localization of cortical markers and kinetochore proteins might be more random at higher temperature, leading to random segregation.

## 4. Material and methods

### 4.1. Yeast strains, growth conditions and medium

The strains used in this study and their genotypes are listed in electronic supplementary material, table S6. Strain construction and maintenance were as described [58]. The cells were grown in yeast extract medium (YES) at 25°C. The cells were synchronized in G1 phase by incubating a *cdc10-M17* mutant at 36°C for 3 h before releasing them into the cell cycle at 25°C.

### 4.2. Cldu and IdU incorporation and detection

Cells grown in YES containing 1 µM oestradiol to keep Sep1 active were synchronized in G1. Oestradiol was removed before release by washing the cells three times with equal volumes of YES. The cells were then released into YES containing 95 µM 5-Chloro-2′-deoxyuridine (CldU) and incubated for 1 h before CldU was removed by washing the cells three times with equal volumes of YES. The cells were then incubated for 2 h before 95 µM iodo-deoxyuridine (IdU) was added and kept in the medium for 1 h. The cells were washed three times with YES medium to remove IdU. The cells were incubated until septa were formed and fixed in 70% ethanol, washed once with PBS and treated with 1 mg ml^−1^ zymolyase 20 T (Sunrise Science Products) for 20 min at 36°C. The cells were treated with 4 M HCl for 10 min, washed three times with PBS, and incubated for 1 h in PBS, 10% fetal calf serum (FCS) and 0.05% Tween-20. Primary antibody against CldU (BU/175, Abcam, cat. no. 7384) or IdU (BU44, Becton Dickinson, cat. no. 347580) was added at a dilution of 1 : 2000 or 1 : 1000, respectively, and the cells were incubated overnight at 4°C on a rotating wheel. The next day, the cells were washed three times with PBS. Secondary anti-rat IgG : Alexa Fluor 568 (Invitrogen cat. no. A11077) or secondary anti-mouse IgG1 : FITC (AbD Serotec cat. no. STAR132F) was added to a dilution of 1 : 250. After incubation for 2 h at room temperature, the cells were washed three times with PBS. For analyses by immunoflourescence microscopy, cells were mounted on poly-l-lysine microscope slides, dried and viewed in the presence of 0.2 µg ml^−1^ 4′,6-diamidino-2-phenylindole (DAPI). Images were collected by a Confocal/Deltavision or Leica CTR DM6000 microscope with a Leica DFC350FX camera.

### 4.3. Edu incorporation and detection

Cells grown in YES containing 1 µM oestradiol were synchronized in G1 and released in the presence of 10 µM 5-ethynyl-2′-deoxyuridine (EdU). After 1 h, the analogue and the oestradiol was removed from the medium by washing three times with equal volumes of YES. The cells were incubated and fixed in 70% ethanol at the time points indicated, washed once with PBS containing 2% FCS (Gibco) and 0.05% Tween-20 (Sigma-Aldrich), and treated with 1 mg ml^−1^ zymolyase 20 T (Sunrise Science Products) for 20 min at 36°C. The cells were washed once with PBS and permeabilized with 1% triton for 1 min. For EdU detection, the Click-IT EdU Alexa Flour 488/555 kit (Life Science) was used as described by the manufacturer. For analyses by immunoflourescence microscopy, cells were mounted on poly-l-lysine microscope slides, dried and viewed in the presence of 0.2 µg ml^−1^ 4′,6-diamidino-2-phenylindole (DAPI). Images were collected by a Deltavision microscope.

### 4.4. Lactose gradient synchronization and Cdc7 : Myc immunofluorescence

Cells grown in YES containing 1 µM oestradiol were synchronized in G2 by lactose gradient centrifugation. Two hours after synchronization, before the cells enter S phase, 10 µM EdU was added and kept in the medium for 1 h. The EdU and oestradiol was removed by washing three times with equal volumes of YES and the cells were incubated for 6–7 h until four-celled mycelia were formed. The cells were fixed in 100% methanol and processed as described above. Primary Myc-antibody (Upstate cat. no. 50-171-788) was added at a dilution of 1 : 200 and the cells were incubated at 4°C over night at a rotating wheel. The next day, the EdU Click-IT reaction Alexa Fluor 488 was performed as described by the manufacturer. The cells were then incubated with secondary mouse Cy3 antibody (cat. no. C2181-ML, Sigma). The cells were incubated for 5 min with 0.2 µg DAPI in PBS, washed three times with PBS and mounted in glycerol-PPD slides and imaged using a Deltavision microscope.

### 4.5. Fluorescence microscopy

Fluorescence micrographs were acquired using a Deltavision OMX V4 microscope or a Deltavision pDV microscope (GE Healthcare, Issaquah, WA). Both instruments were equipped with cooled CCD or sCMOS cameras, a 60× 1.42 NA objective (Olympus) and a solid-state light source. Z-stacks (40 sections with a spacing of 0.125 µM) were collected to ensure that all parts of the nucleus could be measured. Raw images were deconvolved using Softworx software and 3D iterative deconvolution logarithm and sum-projected for measuring the nuclear fluorescence intensity. Measurements were performed using ImageJ (Rasband, W.S., ImageJ, US National Institutes of Health, Bethesda, Maryland, USA, http://imagej.nih.gov/ij/, 1997–2012.)

### 4.6. Detection of harlequin chromosomes using structured illumination microscopy

Cells grown in YES supplemented with 1 µM oestradiol were synchronized in G1 and released in the presence of 10 µM EdU. After 1 h the analogue and the oestradiol were removed from the medium by washing 3 times with equal volumes of YES. A 25 µg ml^−1^ CBZ was added before the third mitosis after release and the cells were fixed, processed and EdU detection was performed as described above. The cells were incubated for 5 min with 0.2 µg DAPI in PBS, washed three times with PBS and mounted on 1% agarose slides. Three-dimensional SIM imaging was performed on Deltavision OMX V4 microscope equipped with an Olympus × 60 NA 1.42 objective and three PCO.edge sCMOS cameras and 405 nm, 488 nm, 568 nm and 647 nm laser lines. Cells were illuminated with a grid pattern and for each image plane, 15 raw images (five phases and three rotations) were acquired. Super-resolution images were reconstructed from the raw image files aligned and projected using Softworx software (Applied Precision, GE Healthcare).

## Data accessibility

All data are presented in the figures, tables or in electronic supplementary material, tables.

The data are provided in electronic supplementary material [59].

## References

[1] Horvitz HR, Herskowitz I. 1992 Mechanisms of asymmetric cell division: two Bs or not two Bs, that is the question. Cell **68**, 237-255. (10.1016/0092-8674(92)90468-R) 1733500

[2] Yadlapalli S, Yamashita YM. 2013 Chromosome-specific nonrandom sister chromatid segregation during stem-cell division. Nature **498**, 251-254. (10.1038/nature12106) 23644460 PMC3711665

[3] Shapiro JA. 1998 Thinking about bacterial populations as multicellular organisms. Annu. Rev. Microbiol. **52**, 81-104. (10.1146/annurev.micro.52.1.81) 9891794

[4] Macara IG, Mili S. 2008 Polarity and differential inheritance—universal attributes of life? Cell **135**, 801-812. (10.1016/j.cell.2008.11.006) 19041746 PMC2844324

[5] Neumüller RA, Knoblich JA. 2009 Dividing cellular asymmetry: asymmetric cell division and its implications for stem cells and cancer. Genes Dev. **23**, 2675-2699. (10.1101/gad.1850809) 19952104 PMC2788323

[6] Gonsalvez GB, Urbinati CR, Long RM. 2005 RNA localization in yeast: moving towards a mechanism. Biol. Cell **97**, 75-86. (10.1042/BC20040066) 15601259

[7] White MA, Eykelenboom JK, Lopez-Vernaza MA, Wilson E, Leach DR. 2008 Non-random segregation of sister chromosomes in *Escherichia coli*. Nature **455**, 1248-1250. (10.1038/nature07282) 18972020

[8] Rocheteau P, Gayraud-Morel B, Siegl-Cachedenier I, Blasco Maria A, Tajbakhsh S. 2012 A subpopulation of adult skeletal muscle stem cells retains all template DNA strands after cell division. Cell **148**, 112-125. (10.1016/j.cell.2011.11.049) 22265406

[9] Cairns J. 1975 Mutation selection and the natural history of cancer. Nature **255**, 197-200. (10.1038/255197a0) 1143315

[10] Kiel MJ et al. 2007 Haematopoietic stem cells do not asymmetrically segregate chromosomes or retain BrdU. Nature **449**, 238-242. (10.1038/nature06115) 17728714 PMC2633872

[11] Verdoodt F *et al*. 2012 Stem cells propagate their DNA by random segregation in the flatworm *Macrostomum lignano*. PLoS ONE **7**, e30227. (10.1371/journal.pone.0030227) 22276162 PMC3261893

[12] Sotiropoulou PA, Candi A, Blanpain C. 2008 The majority of multipotent epidermal stem cells do not protect their genome by asymmetrical chromosome segregation. Stem Cells **26**, 2964-2973. (10.1634/stemcells.2008-0634) 18772311

[13] Falconer E et al. 2010 Identification of sister chromatids by DNA template strand sequences. Nature **463**, 93-97. (10.1038/nature08644) 20016487 PMC3757939

[14] Schepers AG, Vries R, van den Born M, van de Wetering M, Clevers H. 2011 Lgr5 intestinal stem cells have high telomerase activity and randomly segregate their chromosomes. Embo. J. **30**, 1104-1109. (10.1038/emboj.2011.26) 21297579 PMC3061032

[15] Quyn AJ et al. 2010 Spindle orientation bias in gut epithelial stem cell compartments is lost in precancerous tissue. Cell. Stem. Cell. **6**, 175-181. (10.1016/j.stem.2009.12.007) 20144789

[16] Potten CS, Owen G, Booth D. 2002 Intestinal stem cells protect their genome by selective segregation of template DNA strands. J. Cell. Sci. **115**, 2381-2388. (10.1242/jcs.115.11.2381) 12006622

[17] Escobar M *et al*. 2011 Intestinal epithelial stem cells do not protect their genome by asymmetric chromosome segregation. Nat. Commun. **2**, 258. (10.1038/ncomms1260) 21448157 PMC3072071

[18] Tajbakhsh S. 2008 Stem cell identity and template DNA strand segregation. Curr. Opin. Cell Biol. **20**, 716-722. (10.1016/j.ceb.2008.10.004) 18996191

[19] Lansdorp PM. 2007 Immortal strands? Give me a break. Cell **129**, 1244-1247. (10.1016/j.cell.2007.06.017) 17604711

[20] Mitchison JM, Nurse P. 1985 Growth in cell length in the fission yeast *Schizosaccharomyces pombe*. J. Cell. Sci. **75**, 357-376. (10.1242/jcs.75.1.357) 4044680

[21] Mata J, Nurse P. 1998 Discovering the poles in yeast. Trends Cell. Biol. **8**, 163-167. (10.1016/S0962-8924(98)01224-0) 9695831

[22] Simanis V. 2015 Pombe's thirteen—control of fission yeast cell division by the septation initiation network. J Cell. Sci. **128**, 1465-1474. 25690009 10.1242/jcs.094821

[23] Feoktistova A et al. 2012 The fission yeast septation initiation network (SIN) kinase, Sid2, is required for SIN asymmetry and regulates the SIN scaffold, Cdc11. Mol. Biol. Cell. **23**, 1636-1645. (10.1091/mbc.e11-09-0792) 22419817 PMC3338431

[24] Bajpai A *et al*. 2013 Dynamics of SIN asymmetry establishment. PLoS Comput. Biol. **9**, e1003147. (10.1371/journal.pcbi.1003147) 23874188 PMC3708865

[25] Grallert A, Krapp A, Bagley S, Simanis V, Hagan IM. 2004 Recruitment of NIMA kinase shows that maturation of the *S*. pombe *spindle-pole body occurs over consecutive cell cycles and reveals a role for NIMA in modulating SIN activity*. Genes Dev. **18**, 1007-1021. (10.1101/gad.296204) 15132994 PMC406291

[26] Sohrmann M, Schmidt S, Hagan I, Simanis V. 1998 Asymmetric segregation on spindle poles of the *Schizosaccharomyces pombe* septum-inducing protein kinase Cdc7p. Genes Dev. **12**, 84-94. (10.1101/gad.12.1.84) 9420333 PMC316397

[27] Cerutti L, Simanis V. 1999 Asymmetry of the spindle pole bodies and spg1p GAP segregation during mitosis in fission yeast. J. Cell Sci. **112**, 2313-2321. (10.1242/jcs.112.14.2313) 10381387

[28] Klar AJ. 1987 Differentiated parental DNA strands confer developmental asymmetry on daughter cells in fission yeast. Nature **326**, 466-470. (10.1038/326466a0) 3561486

[29] Yu C, Bonaduce MJ, Klar AJ. 2013 Defining the epigenetic mechanism of asymmetric cell division of *Schizosaccharomyces japonicus* yeast. Genetics **193**, 85-94. (10.1534/genetics.112.146233) 23150598 PMC3527257

[30] Klar AJ. 1994 A model for specification of the left-right axis in vertebrates. Trends Genet. **10**, 392-396. (10.1016/0168-9525(94)90055-8) 7809944

[31] Sipiczki M, Grallert B, Miklos I. 1993 Mycelial and syncytial growth in *Schizosaccharomyces pombe* induced by novel septation mutations. J. Cell. Sci. **104**, 485-493. (10.1242/jcs.104.2.485) 8505375

[32] Boe CA *et al*. 2008 Rapid regulation of protein activity in fission yeast. BMC Cell Biol. **9**, 23. (10.1186/1471-2121-9-23) 18457584 PMC2408571

[33] Hodson JA, Bailis JM, Forsburg SL. 2003 Efficient labeling of fission yeast *Schizosaccharomyces pombe* with thymidine and BUdR. Nucleic Acids Res. **31**, e134. (10.1093/nar/gng134) 14576334 PMC275491

[34] Morris SM. 1991 The genetic toxicology of 5-bromodeoxyuridine in mammalian cells. Mutat. Res. **258**, 161-188. (10.1016/0165-1110(91)90007-I) 1881403

[35] Anda S, Boye E, Grallert B. 2014 Cell-cycle analyses using thymidine analogues in fission yeast. PLoS ONE **9**, e88629. (10.1371/journal.pone.0088629) 24551125 PMC3923809

[36] Davidson MB et al. 2012 Endogenous DNA replication stress results in expansion of dNTP pools and a mutator phenotype. Embo. J. **31**, 895-907. (10.1038/emboj.2011.485) 22234187 PMC3280564

[37] Håkansson P, Dahl L, Chilkova O, Domkin V, Thelander L. 2006 The *Schizosaccharomyces pombe* replication inhibitor Spd1 regulates ribonucleotide reductase activity and dNTPs by binding to the large Cdc22 subunit. J. Biol. Chem. **281**, 1778-1783. (10.1074/jbc.M511716200) 16317005

[38] Kumar D et al. 2011 Mechanisms of mutagenesis in vivo due to imbalanced dNTP pools. Nucleic Acids Res. **39**, 1360-1371. (10.1093/nar/gkq829) 20961955 PMC3045583

[39] Lasken RS, Goodman MF. 1984 The biochemical basis of 5-bromouracil-induced mutagenesis. Heteroduplex base mispairs involving bromouracil in G·C → A·T and A·T → G·C mutational pathways. J. Biol. Chem. **259**, 11 491-11 495. (10.1016/S0021-9258(18)90888-4) 6088545

[40] Sivakumar S, Porter-Goff M, Patel PK, Benoit K, Rhind N. 2004 In vivo labeling of fission yeast DNA with thymidine and thymidine analogs. Methods **33**, 213-219. (10.1016/j.ymeth.2003.11.016) 15157888 PMC5074384

[41] Smith CL et al. 1987 An electrophoretic karyotype for *Schizosaccharomyces pombe* by pulsed field gel electrophoresis. Nucleic Acids Res. **15**, 4481-4489. (10.1093/nar/15.11.4481) 3295780 PMC340875

[42] Klar AS, Bonaduce M. 2013 Unbiased segregation of fission yeast chromosome 2 strands to daughter cells. Chromosome Res. **21**, 297-309. (10.1007/s10577-013-9352-1) 23681661 PMC6959520

[43] Coelho M et al. 2013 Fission yeast does not age under favorable conditions, but does so after stress. Curr. Biol. **23**, 1844-1852. (10.1016/j.cub.2013.07.084) 24035542 PMC4620659

[44] Zuin A et al. 2010 Lifespan extension by calorie restriction relies on the Sty1 MAP kinase stress pathway. EMBO J. **29**, 981-991. (10.1038/emboj.2009.407) 20075862 PMC2837171

[45] Baldi S, Bolognesi A, Meinema AC, Barral Y. 2017 Heat stress promotes longevity in budding yeast by relaxing the confinement of age-promoting factors in the mother cell. Elife 6. (10.7554/eLife.28329) PMC5771669 29283340

[46] Meuth M, Green H. 1974 Induction of a deoxycytidineless state in cultured mammalian cells by bromodeoxyuridine. Cell **2**, 109-112. (10.1016/0092-8674(74)90099-3) 4477047

[47] Sabatinos SA, Green MD, Forsburg SL. 2012 Continued DNA synthesis in replication checkpoint mutants leads to fork collapse. Mol. Cell. Biol. **32**, 4986-4997. (10.1128/MCB.01060-12) 23045396 PMC3510540

[48] Falconer E, Chavez E, Henderson A, Lansdorp PM. 2010 Chromosome orientation fluorescence in situ hybridization to study sister chromatid segregation in vivo. Nat. Protoc. **5**, 1362-1377. (10.1038/nprot.2010.102) 20595964 PMC3771506

[49] Armakolas A, Koutsilieris M, Klar AJS. 2010 Discovery of the mitotic selective chromatid segregation phenomenon and its implications for vertebrate development. Curr. Opin. Cell Biol. **22**, 81-87. (10.1016/j.ceb.2009.11.006) 20022232 PMC7241865

[50] Klar AJ. 1990 The developmental fate of fission yeast cells is determined by the pattern of inheritance of parental and grandparental DNA strands. EMBO J. **9**, 1407-1415. (10.1002/j.1460-2075.1990.tb08256.x) 2328720 PMC551827

[51] Dalgaard JZ, Klar AJ. 1999 Orientation of DNA replication establishes mating-type switching pattern in *S*. pombe. Nature **400**, 181-184. (10.1038/22139) 10408447

[52] Yamashita YM, Mahowald AP, Perlin JR, Fuller MT. 2007 Asymmetric inheritance of mother versus daughter centrosome in stem cell division. Science **315**, 518-521. (10.1126/science.1134910) 17255513 PMC2563045

[53] Pereira G, Tanaka TU, Nasmyth K, Schiebel E. 2001 Modes of spindle pole body inheritance and segregation of the Bfa1p-Bub2p checkpoint protein complex. EMBO J. **20**, 6359-6370. (10.1093/emboj/20.22.6359) 11707407 PMC125717

[54] Feierbach B, Chang F. 2001 Roles of the fission yeast formin for3p in cell polarity, actin cable formation and symmetric cell division. Curr. Biol. **11**, 1656-1665. (10.1016/S0960-9822(01)00525-5) 11696322

[55] Mikule K et al. 2007 Loss of centrosome integrity induces p38-p53-p21-dependent G1-S arrest. Nat. Cell. Biol. **9**, 160-170. (10.1038/ncb1529) 17330329

[56] Bornens M, Piel M. 2002 Centrosome inheritance: birthright or the privilege of maturity? *Curr*. Biol. **12**, R71-R73. (10.1016/S0960-9822(01)00678-9) 11818084

[57] Thorpe PH, Bruno J, Rothstein R. 2009 Kinetochore asymmetry defines a single yeast lineage. Proc. Natl Acad. Sci. USA **106**, 6673-6678. (10.1073/pnas.0811248106) 19346480 PMC2672522

[58] Moreno S, Klar A, Nurse P. 1991 Molecular genetic analysis of fission yeast *Schizosaccharomyces pombe*. Methods Enzymol. **194**, 795-823. (10.1016/0076-6879(91)94059-L) 2005825

[59] Anda S, Boye E, Schink KO, Grallert B. 2021 Cosegregation of asymmetric features during cell division. Figshare. 10.1098/rsob.210116 PMC8331232 34343465

